# MUPS Tableting—Comparison between Crospovidone and Microcrystalline Cellulose Core Pellets

**DOI:** 10.3390/pharmaceutics14122812

**Published:** 2022-12-15

**Authors:** Daniel Robin Thio, Paul Wan Sia Heng, Lai Wah Chan

**Affiliations:** GEA-NUS Pharmaceutical Processing Research Laboratory, Department of Pharmacy, National University of Singapore, Singapore 117543, Singapore

**Keywords:** MUPS tablet, pellet core, spheronization aid, ethylcellulose, pellet coat damage, crospovidone, microcrystalline cellulose, compaction energy

## Abstract

Multi-unit pellet system (MUPS) tablets were fabricated by compacting drug-loaded pellets of either crospovidone or microcrystalline cellulose core. These pellets were produced by extrusion-spheronization and coated with ethylcellulose (EC) for a sustained drug release function. Coat damage due to the MUPS tableting process could undermine the sustained release function of the EC-coated pellets. Deformability of the pellet core is a factor that can impact the extent of pellet coat damage. Thus, this study was designed to evaluate the relative performance of drug-loaded pellets prepared with either microcrystalline cellulose (MCC) or crospovidone (XPVP) as a spheronization aid and were comparatively evaluated for their ability to withstand EC pellet coat damage when compacted. These pellets were tableted at various compaction pressures and pellet volume fractions. The extent of pellet coat damage was assessed by the change in drug release after compaction. The findings from this study demonstrated that pellets spheronized with XPVP had slightly less favorable physical properties and experienced comparatively more pellet coat damage than the pellets with MCC. However, MUPS tablets of reasonable quality could successfully be produced from pellets with XPVP, albeit their performance did not match that of vastly mechanically stronger pellets with MCC at higher compaction pressure.

## 1. Introduction

Multi-unit pellet system (MUPS) tablets consist of drug-loaded pellets compacted with fillers that possess some capability to provide cushioning or damage mitigating support. These drug-loaded pellets can be fabricated in a multitude of ways. The most common method is by extrusion-spheronization, as it is regarded as a robust and reproducible process, capable of fabricating high-quality spheroids of ample mechanical strength and narrow size distribution [1,2]. Other pellet production methods include suspension/solution layering, spray congealing, rotary processing, high/low shear granulation, and powder layering [3,4]. Pellets containing drug can be coated with a semipermeable polymeric coat for a sustained drug release function, which may be impaired during the MUPS tableting process [5,6,7]. One of the factors affecting the degree of pellet coat damage, and hence the sustained release function of the MUPS tablet, is the physical properties of the pellet core [8,9,10]. The extent of pellet coat damage must be mitigated to successfully formulate MUPS tablets with relatively unimpaired sustained drug release function when compared with the uncompacted pellets. One among many strategies to mitigate pellet coat damage is to use materials amenable to the extrusion-spheronization process but with different physical properties so that deformability of the resultant pellet cores could also be different. The profile of these cores as coated pellets to compaction damage remains to be investigated. In cases of excipient-drug incompatibility, an alternative excipient is needed and therefore the deformative property of the pellet cores also requires attention, as it could affect the extent of compaction-related coat damage.

In order to understand how different materials can affect the extrusion-spheronization process, the steps of the process must be well understood. These steps include mixing, wet massing, extruding, spheronizing, and drying. Briefly, there is a multitude of factors that can influence the end quality of the pellets, such as the formulation (excipients, granulating liquid [11,12], drug), equipment (mixer, granulator, extruder with screen, spheronizer with friction plate, dryer), and process parameters (extrusion rate, spheronization speed and time, material loading, drying method) [13,14,15,16]. After wet massing, the primary particles coalesce into three-phase air–water–solid aggregates that are extruded and spheronized [17].

During the extrusion-spheronization process, the material has to be optimally wetted to effectively form good-quality pellets [18]. The wetted mass for spheronization forms a cohesive yet plastic-enough mass that also remains homogenous throughout the process [19]. There are specific requirements of moisture in the wetted mass for both extrusion and spheronization. However, many materials, when wetted, do not possess the characteristics required to work effectively for both processes [20]. Formulators must strike a precise balance between material plasticity and brittleness of the extrudate to produce spherical pellets. Understandably, most wetted materials do not exert the necessary properties and therefore necessitate the inclusion of a spheronization aid [14]. The most commonly used spheronization aid is microcrystalline cellulose (MCC) due to its favorable cohesiveness, rheological properties, and plasticity, which enable the formation of uniform, strong, and spherical pellets [21,22,23]. MCC is regarded as an excellent spheronization aid due to its high internal porosity and large surface area, which can retain adequate moisture for effective lubrication during the extrusion process [1,20]. Subsequently, moisture retained in the MCC microfibrils is critical to forming a cohesive mass and providing plasticity for reshaping extrudate fragments into pellets during spheronization [24,25,26]. Although MCC can be an effective spheronization aid in most instances, it has some limitations. MCC has been reported to be incompatible with various drugs [27,28,29,30,31,32,33] and can adsorb poorly water-soluble drugs such as ketotifen, famotidine, and phenothiazine [27,28]. The adsorption of drug to MCC can undesirably impair the intended drug release profile and thus limit the effectiveness of the sustained release dosage form [14,34].

Recent efforts into finding an alternative spheronization aid to MCC have been fruitful [21]. Cross-linked polyvinylpyrrolidone or crospovidone (XPVP) was successfully used as a spheronization aid to form pellets [2,35,36,37]. The suitability of XPVP for drug-loaded pellets with a sustained release coat in MUPS tablets has not been extensively studied. However, XPVP-based pellets containing some drugs, such as caffeine, paracetamol, fexofenadine hydrochloride, hydrochlorothiazide, and spironolactone, have been reported [29,31,32,33,38]. Researchers used a Box–Behnken design of experiments to assess the quality of pellets spheronized with XPVP or MCC and found that pellets spheronized using XPVP were generally weaker [2]. This effect was attributed to the lower ability of XPVP to absorb water compared to MCC. It was hypothesized that the fibrous and needle-shape structure of MCC allows for stronger binding through mechanical interlocking with the bulk material and increased retention of water. Furthermore, XPVP was comparatively not as effective as MCC due to its narrower water-concentration range where material rheology is suitable for extrusion-spheronization. It was reported that the factors most critical to the quality of pellets spheronized with XPVP were the XPVP and water concentrations. Regardless, the quality of these pellets was comparable to those spheronized with MCC when the XPVP concentration, water concentration, spheronization time, and spheronization speed were set at their highest levels. Unlike other materials, XPVP and MCC do not require an additional binder before acting as a spheronization aid [2].

The aim of this study is to further explore the use of XPVP as a spheronization aid for drug-loaded pellets coated with a sustained release layer. Furthermore, the main objective is to compare the performance of pellets spheronized with XPVP to those with MCC in terms of pellet coat damage due to compaction. Two parameters were used as the basis of this comparison in performance. The first parameter was the compaction pressure and the second the MUPS tablet pellet volume fraction or the number of pellets contained in the MUPS tablet. Subsequently, the damage to the sustained release ethylcellulose (EC) coat was assessed through drug dissolution. A faster drug release than uncompacted pellets would indicate a higher degree of EC pellet coat damage [7,39,40,41]. In the present work, it was found that MUPS tablets can be successfully made with XPVP core pellets, but their performance against pellet coat damage was slightly inferior compared to the MCC core pellets. It was postulated that comparatively greater plastic deformation of the XPVP pellet due to its weaker mechanical strength ultimately exerted greater stress on the EC pellet coat, thereby causing more pellet coat damage during compaction.

## 2. Materials and Methods

### 2.1. Materials

Pellets were fabricated with α-lactose monohydrate (Granulac 200^®^, Meggle Pharma, Wasserburg am Inn, Germany), MCC (Ceolus^®^ PH-101, Asahi Kasei, Tokyo, Japan) or XPVP (Kollidon CL-M^®^, BASF, Ludwigshafen, Germany), and metformin hydrochloride (MET; Granules India, Hyderabad, India). The sustained release layering material consisted of ethylcellulose (EC) dispersion (Aquacoat^®^ ECD, FMC Biopolymer, Philadelphia, PA, USA), triethyl citrate (Merck Schuchardt OHG, Hohenbrunn, Germany) as a plasticizer, and talc powder (Guangxi Longguang Talc Development Co., Ltd., Guilin, China) as an anti-adherent. MCC was also the tablet filler with sodium starch glycolate (Explotab^®^, JRS Pharma, Pirna, Germany). Purified water was used as the dissolution and disintegration media.

### 2.2. Preparation of Coated Pellets

#### 2.2.1. Fabrication of MET-Loaded Pellets Containing Either MCC or XPVP Pellets

The MET-loaded pellets were fabricated using extrusion-spheronization with MCC or XPVP, lactose, and MET in a ratio of 1:1:3 by weight. The ingredients were weighed out and blended in a cube mixer (AR400E, Erweka, Langen, Germany) at 100 rpm for 20 min. The powder blend was then transferred into a planetary mixer (Kenwood Major, Kenwood Limited, Havant, UK), mixed, and wetted over 5 min with deionized water equivalent to 25%, *w*/*w* of the dry powder blend while mixing. The resultant wetted mass was extruded through a 1 mm aperture screen using a radial extruder (E140, GEA, Eastleigh, UK). The extrudate collected was spheronized (S320, GEA, Eastleigh, UK) on a 320 mm cross-hatched frictional plate rotated at 498 rpm for 10 min. The pellets formed were collected and oven dried at 60 °C for about 12 h. The dried pellets were then sieved (Endecotts, London, UK) to obtain a fraction of 0.85–1.4 mm. Pellets spheronized with MCC or XPVP are hereafter referred to as MCC or XPVP pellets, respectively.

#### 2.2.2. Ethylcellulose Coating of MET-Loaded Pellets

MET-loaded pellets (250 g) were transferred into a Wurster fluid bed coater (Strea-1, GEA, Eastleigh, UK) and spray coated with EC to a final coat weight gain of 12%, *w*/*w*. The coating-process parameters are listed in Table 1 and the coating medium consisted of 39.22% *w*/*w* EC dispersion, 2.35% *w*/*w* triethyl citrate, 5.88% *w*/*w* talc, and 52.55% *w*/*w* deionized water. Under homogenization (L4R, Silverson, Chesham, UK), triethyl citrate, talc, and deionized water were mixed for 10 min. The mixture was then added to the EC dispersion and stirred with an overhead stirrer for 40 min before use for coating. The coated pellets were collected and sieved (Endecotts, London, UK), and only the fraction of 0.85–1 mm was used.

### 2.3. Characterization of Coated Pellets

#### 2.3.1. Determination of Individual Pellet Weight

Individual pellet weight was measured by randomly selecting 100 pellets from each batch and determining their total weight using a precision balance (AG 135, Mettler Toledo, Columbus, OH, USA). The individual pellet weight was then calculated by dividing the total weight by 100. At least 3 replicates were carried out.

#### 2.3.2. Determination of Pellet Crushing Strength

The crushing strength of individual EC-coated pellets was determined using a universal tester (EZ Test SM-100, Shimadzu Corporation, Kyoto, Japan) with a 100 g force sensor. Equivalent-sized pellets were obtained by passing the pellets through a 1 mm sieve (Endecotts, London, UK). The pellets were then lightly re-sieved using the same 1 mm sieve and those that were trapped on the mesh were collected for crushing strength testing. A single pellet was placed between two compression plates of the universal tester and the maximum load (F_m_) required to break the pellet at a compression rate of 0.5 mm/min was recorded. For each batch, at least 30 pellets were randomly chosen and tested. The pellet crushing strength was then calculated using the following equation:

(1) $$\mathrm{Crushing}\;\mathrm{strength}\;\left(\mathrm{MPa}\right)=\frac{{4F}_{m}}{{\pi d}^{2}}$$

where d is the aperture size of the sieve (Endecotts, London, UK), representing the diameter of the pellets tested [42].

#### 2.3.3. Evaluation of Pellet Shape

Pellet shape was assessed using a stereomicroscope (SZ61, Olympus Corporation, Tokyo, Japan) by acquiring images of at least 600 randomly selected pellets using an attached digital microscope camera (DP71, Olympus Corporation, Tokyo, Japan). These images were subsequently analyzed with an imaging software (Image-Pro, Version 6.3, Media Cybernetics, Rockville, MD, USA) to determine the pellet aspect ratio and roundness. The pellet aspect ratio measures the elongation of the pellets and was calculated using the following equation:

(2) $$\mathrm{Aspect}\;\mathrm{ratio}=\frac{l}{b}$$

where *l* is the is the length of a straight line connecting the two most distant points of the two-dimensional pellet outline and *b* is the breadth perpendicular to *l*.

The pellet roundness describes the sphericity of the pellets and was calculated using the following equation:

(3) $$\mathrm{Roundness}=\frac{P^{2}}{4\pi A\;}$$

where P and A are the perimeter and the area of the pellet, respectively. A pellet roundness closer to unity indicates a perfect circle, which may be desirable for pharmaceutical pellets of good quality.

#### 2.3.4. Evaluation of Pellet Size and Size Distribution

The pellet sizes were assessed using an optical particle sizer (Eyecon 3D Particle Characterizer, Innopharma, Dublin, Ireland) and at least 600 randomly selected pellets were sized. A cumulative undersize plot was generated where D10, D50, and D90 represent the pellet sizes at the 10th, 50th, and 90th cumulative percentile, respectively. The mean particle size (MPS) was calculated by averaging the pellet sizes. The pellet span was calculated using the following equation:

(4) $$\mathrm{Span}=\frac{(D90\;-\;D10)}{D50}$$

A lower span indicates a narrower pellet size distribution, which would be desirable for the effective production of pharmaceutical pellets.

#### 2.3.5. Determination of Pellet True Density and True Volume

A helium-displacement pycnometer (Penta-Pycnometer, Anton Paar, Graz, Austria) was used to determine the true density p_t_ of coated pellets loaded with MET. At least three replicates were carried out and the results were averaged. The true volume V_p_ of pellets was calculated as reported previously [5,40,43] using the following equation:

(5) $$V_{p}=\frac{\mathrm{pellet}\;\mathrm{sample}\;\mathrm{mass}}{p_{t}}$$

### 2.4. Tableting of Coated Pellets

#### 2.4.1. Tableting Using a Compaction Simulator

A compaction simulator (Styl’One Evolution, Medelpharm, Beynost, France) was used to fabricate the MUPS tablets. From the compaction profile, using the control and data-capture software (Analis, Version 2.08.5, Medelpharm, Beynost, France), different energetic parameters were derived as described in previous studies [44,45,46]. Among the different compaction energies captured (compression, rearrangement, plastic, and elastic), the rearrangement energy was of particular interest, as it represents the energy consumed when particles slide over one another without causing excessive deformation [47,48]. The plastic energy represents the energy applied to the stage where particle rearrangement is no longer possible and particle deformation occurs. The elastic energy is the energy of the tablet’s elastic recovery after application of maximum force. The compression energy represents the energy for the formation of the compact and is the sum of the plastic and elastic energy. The ejection energy, representing the energy consumed as the lower punch moves up after compaction to eject the tablet, was also acquired. At least 4 replicates were carried out and the results were averaged.

#### 2.4.2. Tablets Prepared to Evaluate the Effects of Compaction Pressure

The MUPS tablet filler material was prepared by mixing 99% *w*/*w* MCC with 1% *w*/*w* sodium starch glycolate. MUPS tablets were prepared by combining 15 mg of MCC pellets or 15.5 mg of XPVP pellets with filler material to a final weight of 200 mg (±5%). The filler-pellet mass was then mixed and added to the die of a 10 mm flat-faced punches-and-die set mounted in a compaction simulator (Styl’One Evolution, Medelpharm, Beynost, France) set to a fill depth of 8.5 mm. Compaction was carried out at 10, 20, 30, and 40 MPa. Control tablets without pellets were similarly fabricated.

#### 2.4.3. Tablets Prepared to Evaluate the Effects of Pellet Volume Fraction

MUPS tablets were prepared with various pellet volume fractions by adjusting the number of pellets contained therein. The number of pellets and their corresponding weights along with the intended pellet volume fractions are listed in Table 2. An appropriate amount of filler material was added to the weighed amount of pellets to give a final MUPS tablet weight of 200 mg (±5%). The pellet-filler mass was then mixed and compacted as described earlier at 30 MPa to yield tablets of approximately 1 MPa in tensile strength. Control tablets without pellets were similarly fabricated.

### 2.5. Evaluation of MUPS Tablets

#### 2.5.1. Determination of Pellet Volume Fraction

The MUPS tablet apparent volume V_M_ was calculated using the following equation:

(6) $${\;V}_{M}{=\pi \mathrm{hr}}^{2}$$

where r and h are the MUPS tablet radius and height, respectively.

The MUPS tablet pellet volume fraction was calculated as reported previously [5,40] using the following equation:

(7) $$\mathrm{Pellet}\;\mathrm{volume}\;\mathrm{fraction}=\frac{V_{p}}{V_{M}}\times 100$$

A higher pellet volume fraction would increase the numerical presence of pellets in the tablet, which is advantageous for increasing the MUPS tablet dosage but can lead to a higher risk of pellets colliding with other pellets or the tooling.

#### 2.5.2. Tensile Strength Test

The tablet breaking force F was measured after post-compaction recovery of at least 3 days using a tensile strength tester (TBF 1000, Copley Scientific, Nottingham, UK). The tablet tensile strength value was calculated using the following equation:

(8) $$\mathrm{Tensile}\;\mathrm{strength}\;(\mathrm{MPa})=\frac{2F}{\pi \mathrm{Dh}}\;$$

where D is the tablet diameter. Five replicates were carried out and the results were averaged.

#### 2.5.3. Disintegration Test

The disintegration tests were conducted (DT2, Sotax, Westborough, MA, USA) according to the United States Pharmacopoeia (USP) method using purified water maintained at 37 °C. Five replicates were carried out for each condition. The disintegration time is indicative of when the MUPS tablet starts behaving as a multiple-unit preparation and is therefore an important quality attribute of MUPS tablets [49].

#### 2.5.4. Dissolution Test

Uncompacted and tableted pellets were tested for MET release in a USP apparatus 2 (VK7010, Varian, Edison, NJ, USA) with the paddle rotated at 100 rpm in 500 mL of degassed purified water maintained at 37 °C. A 5 mL aliquot sample was withdrawn at 0, 5, 15, 30, 45, and 60 min. After 60 min, the pellets in each vessel were crushed, and the medium was agitated at 220 rpm for another 15 min before the final sampling to determine the total MET available. The concentration of MET was determined spectrophotometrically (UV-5100, Hitachi, Tokyo, Japan) at a wavelength of 232 nm. Of each condition, at least 4 replicated runs were carried out and the results were averaged to establish the dissolution profiles.

#### 2.5.5. Evaluation of Pellet Coat Damage

The mean dissolution time (MDT) represents the drug release rate [50] and can be used as an indicator of pellet coat damage [43,49,51,52,53]. The MDT was calculated using the following equation:

(9) $$\mathrm{MDT}=\frac{\sum_{i=1}^{N}{\bar{t}}_{i}{\Delta M}_{i}}{\sum_{i=1}^{N}{\Delta M}_{i}}$$

where ${\bar{t}}_{i}$ is the midpoint of the time period during which the fraction $\Delta M_{i}$ of the drug has been released from the dosage form at time-point *i* for *N* time points. At least 4 replicates were carried out. MDT values of compacted pellets (MDT_C_) lower than the MDT values of uncompacted pellets (MDT_UC_) indicates a loss in sustained release due to pellet coat damage. The MDT of pellets with different compositions may vary. Therefore, the MDT_C_ was expressed as a percentage (K) of the corresponding MDT_UC_ to permit valid comparison of the extent of pellet coat durability between different pellet types. K was calculated using the following equation:

(10) $$K=\frac{{\mathrm{MDT}}_{C}}{{\mathrm{MDT}}_{\mathrm{UC}}}\times 100\%$$

The K value indicates the integrity of the pellet coat to damage, with K values near 100% indicating a lack of pellet coat damage.

Another pellet coat damage indicator was the MET release rate, which was determined by measuring the difference in the amount of MET released (M) and dividing that difference by the corresponding change in time (T) according to the following equation:

(11) $$\mathrm{MET}\;\mathrm{release}\;\mathrm{rate}=\frac{{(M}_{i+1}\;{\text{–}\;M}_{i})}{{(T}_{i+1\;}\;{\text{–}\;T}_{i})}$$

The initial dissolution rate (IDR) was defined as the first MET release rate and was previously used to assess pellet coat damage [54]. A high IDR would indicate breaches to the EC coat that permit quick dissolution of the water-soluble MET into the medium. High IDR values followed by MET release rate values below those of the uncompacted pellets during the later time periods would indicate a loss in sustained release function.

### 2.6. Scanning Electron Microscopy Imaging

Pellet samples were extracted by gently washing MUPS tablets in a fine mesh strainer using water. Intact MUPS tablets and extracted pellets were then fixed using carbon paste onto cylindrical studs and desiccated for about 12 h. Photomicrographs of the pellet and MUPS tablet surfaces were obtained using a scanning electron microscope (SEM; JSM-6010LV, JEOL, Tokyo, Japan).

### 2.7. Statistical Analysis

When comparing two conditions, an independent-sample *t*-test was used with an alpha level of 0.05 and a significant difference between conditions was denoted by a * in the plots. Regression models (Tableau, Version 2020.3, Tableau Software, Seattle, WA, USA) were fitted using each replicate as a single data point to describe relevant relationships.

## 3. Results

### 3.1. Physical Characterization of Coated Pellets

Pellets were prepared with either MCC or XPVP as a spheronization aid and their physical properties were characterized (Table 3). The results show that the pellets had different physical properties. The values of aspect ratio, span, and mean particle size of the XPVP pellets were higher, albeit not particularly large. Notable were the lower true density and crushing strength of the XPVP pellets.

### 3.2. SEM Imaging

**Figure 1:**
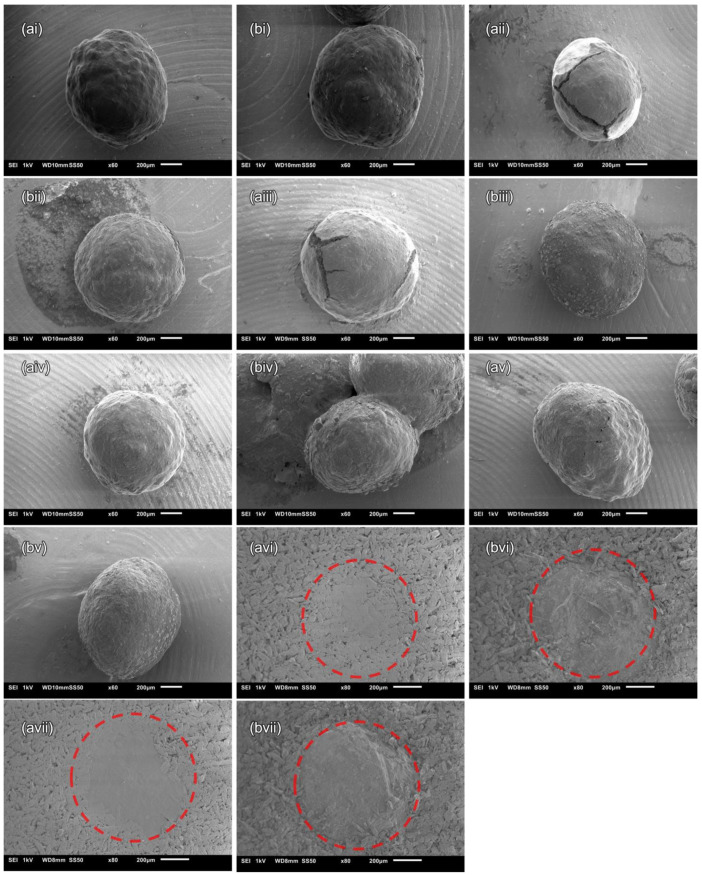
SEM images of (**a**) MCC and (**b**) XPVP pellets that were (**i**) uncompacted or compacted at (**ii**) 10, (**iii**) 20, (**iv**) 30, and (**v**) 40 MPa. Pellets still embedded in the surface of the MUPS tablet compacted at (**vi**) 30 and (**vii**) 40 MPa are encircled by a red dashed line.

The MCC and XPVP pellets were compacted at various pressures and excavated from the MUPS tablets for observation under SEM. Figure 1 depicts the SEM images of the MCC and XPVP pellets. It was exceedingly difficult to extract the pellets from the mechanically stronger MUPS tablets without causing further pellet coat damage. Thus, the filler excipient was gently washed away using water, but some pellets with breached coats, which allowed water to enter the pellet cores, rapidly disintegrated. This effect was especially prevalent for the XPVP pellets, likely because XPVP is a memory polymer that has been reported to disintegrate tablets through rapid volumetric expansion upon water intake [55]. In any case, these coat breaches provided qualitative evidence of compaction-induced pellet coat damage that permits easy and destructive water entry.

### 3.3. Compaction Characterization

The physical nature of the pellet core can affect the MUPS tablet compaction properties [56,57,58] and can thereby also impact the manufacturability of MUPS tablets. The effects of utilizing either MCC or XPVP pellets on the MUPS tablet compaction properties were determined using two methods. The first method involved evaluating the effect of compaction pressure on tablet tensile strength and the second by examining the compaction characteristics derived from the compaction simulator.

#### 3.3.1. Effect of Compaction Pressure on Tablet Tensile Strength

**Figure 2:**
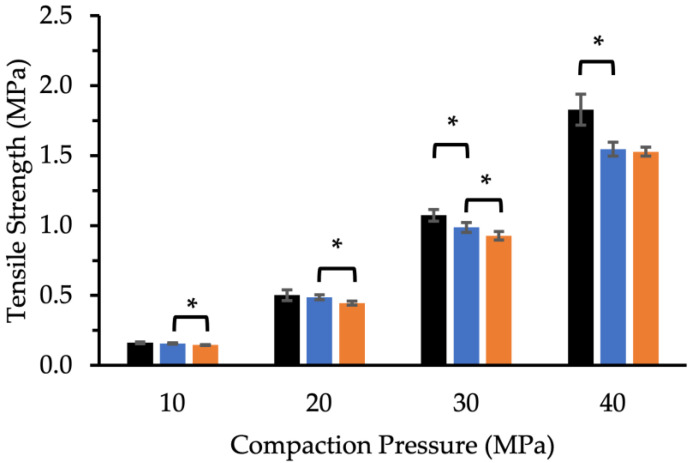
Tensile strength values (*n* = 5) of control tablets (∎) and MUPS tablets compacted at various compaction pressures containing MCC (∎) or XPVP pellets (∎). Error bars indicate the standard deviation and * denotes a significant (*p* < 0.05) difference.

The tensile strength values of MUPS tablets compacted with MCC or XPVP pellets at various compaction pressures were determined (Figure 2). The results indicate that the compaction pressure significantly increased the MUPS tablet tensile strength. For the MUPS tablets prepared at 10, 20, and 30 MPa, the tensile strength values for MUPS tablets containing the MCC pellets were significantly higher (*p* < 0.05) than those with the XPVP pellets. At 40 MPa, there was no significant difference between the tensile strength values of tablets prepared with both pellet types. It was also apparent that the control tablets were generally stronger than the MUPS tablets, especially at higher compaction pressure.

#### 3.3.2. Effect of Compaction Pressure on Compaction Properties

**Figure 3:**
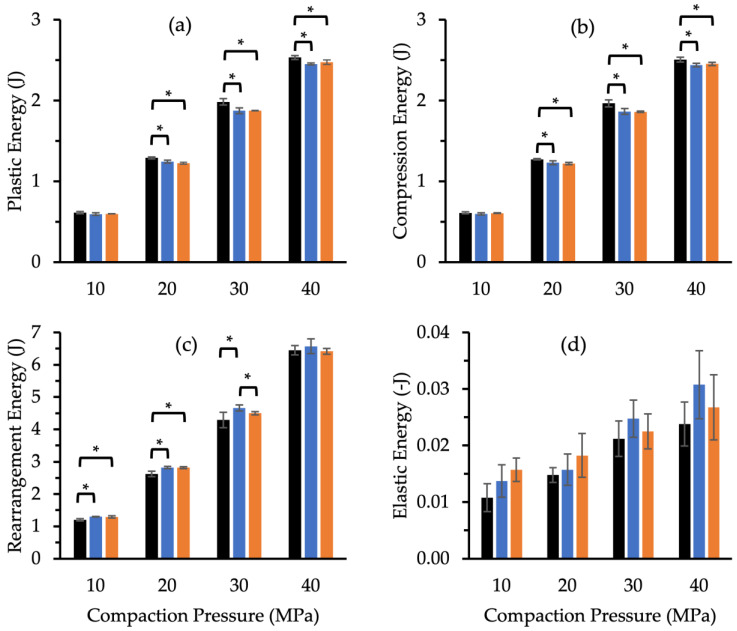
Compaction energy data obtained from the Analis software for control tablets (∎) (*n* = 5) and MUPS tablets (*n* = 4) containing MCC (∎) or XPVP pellets (∎) compacted at various compaction pressures, comprising (**a**) plastic, (**b**) compression, (**c**) rearrangement, and (**d**) elastic energy. Error bars indicate the standard deviation and * denotes a significant (*p* < 0.05) difference.

Increases in compaction pressure and the differing nature of tableting components have been associated with changes in the distribution of energy throughout the compact during tableting [46]. The effect of MUPS tablet compaction with either MCC or XPVP pellets was assessed by deriving the compaction energy profiles (Figure 3) from the compaction force-time profiles at the four compaction pressures. There were significant differences in the compaction energies between the control and MUPS tablets. Moreover, some differences between the compaction of MUPS tablets with MCC and XPVP pellets were observed. Generally, increased compaction pressure led to increases in energetics due to greater energy supplied to the compact by the compaction simulator; hence, comparative assessments were best conducted within each compaction pressure level.

The plastic energies (Figure 3a) were generally higher for the control tablets and not significantly different (*p* > 0.05) between MUPS tablets with MCC or XPVP pellets. Compared to MUPS tablets, the control tablet compression (Figure 3b) and rearrangement (Figure 3c) energies were higher and lower, respectively. Generally, the compression and rearrangement energies for MUPS tablets with MCC or XPVP pellets did not differ much. The rearrangement energy of MUPS tablets with XPVP pellets was significantly lower only at 30 MPa; notwithstanding, MUPS tablets with XPVP pellets consistently exhibited lower rearrangement energies. The rearrangement energies increased with compaction pressure and were generally higher for the MUPS tablets than the control tablets. Although elastic energies (Figure 3d) did not differ significantly (*p* > 0.05), the results hinted that the tableting of pellets led to slightly higher elastic energies, particularly with MUPS tablets containing MCC pellets at a higher compaction pressure.

#### 3.3.3. Effect of Pellet Volume Fraction on Compaction Properties

**Figure 4:**
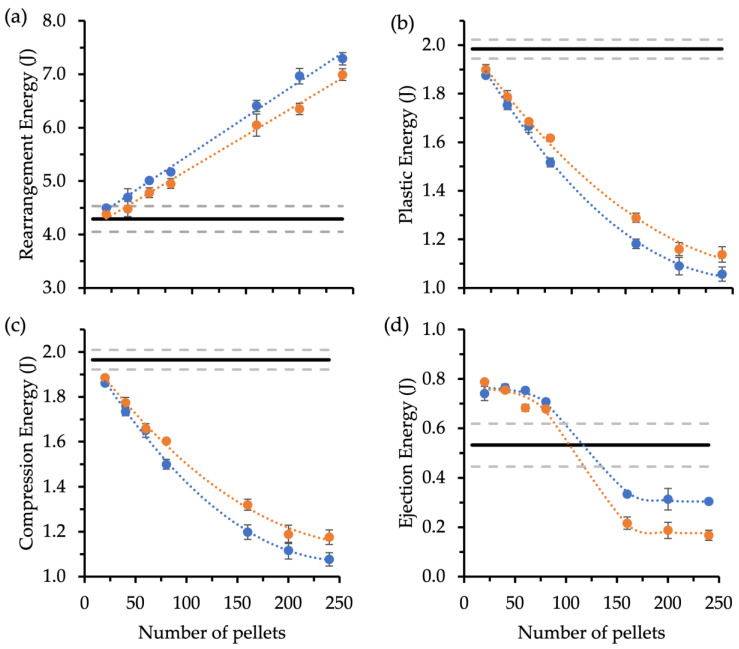
Plots of compaction energies over the number of MCC (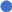) and XPVP (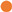) pellets contained in the MUPS tablet (*n* = 4). (**a**) Rearrangement energy, (**b**) plastic energy, (**c**) compression energy, and (**d**) ejection energy. Error bars represent the standard deviation. Trendlines (dotted) were fitted to discern trends. Control tablets (*n* = 5) acted as the baseline, and their compaction properties are indicated by a horizontal line (solid) with the bracketing as standard deviation lines (dashed).

The pellet volume fraction, represented by the number of pellets contained within a MUPS tablet, is expected to affect the compaction properties because increasing the number of pellets would likely decrease the binding ability of filler particles and affect the compaction mechanism of the filler–pellet blend. Nevertheless, increasing the number of pellets or the pellet volume fraction in a MUPS tablet becomes necessary for a MUPS tablet intended to deliver a high drug dose. Therefore, the effects of the pellet nature and number contained within the MUPS tablet on compaction were evaluated by plotting the number of pellets against the compaction energy data (Figure 4). The results indicate that both the pellet type and number played significant roles in the compaction characteristics of the MUPS tablets. Increases in the number of pellets appeared to linearly increase with rearrangement energy, which was generally above that of the control tablets. The rearrangement energy of MUPS tablets with MCC pellets appeared to be greater and increased with a higher number of pellets. The plastic and compression energies appeared to decrease exponentially with the number of pellets and was generally higher for MUPS tablets with XPVP pellets. Both the plastic and compression energies of the MUPS tablets were significantly lower (*p* < 0.05) than those of the control tablets. The ejection energy followed a different relationship altogether, where the ejection energies of MUPS tablets with 80 pellets or less were higher than those of the control tablets. Conversely, the ejection energies of MUPS tablets with 160 pellets or more were significantly lower (*p* < 0.05) than those of control tablets. It was apparent that the MUPS tablets with XPVP pellets had lower ejection energies than those with MCC pellets.

### 3.4. Disintegration

A longer disintegration time may lead to the filler material impeding drug release due to the shielding effect [7]. This shielding effect consists of filler particles physically hindering the release of the drug (MET) from the pellets and can therefore confound the assessment of pellet coat damage. MUPS tablets formulated with MCC were previously reported to be susceptible to the shielding effect due to filler non-disintegration [7]. Hence, the disintegratability of MUPS tablets prepared under the current methodology was assessed to determine whether pellet shielding could occur and confound the pellet coat damage results from the MET release measurements.

**Figure 5:**
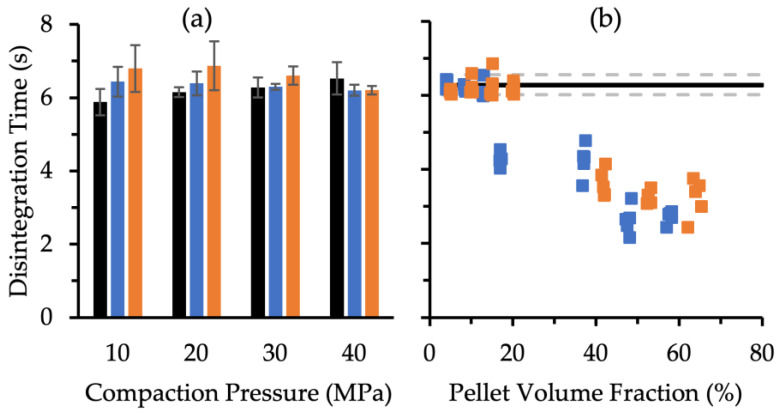
Disintegration times of control tablets (∎) and MUPS tablets prepared with MCC (∎) and XPVP (∎) pellets at various (**a**) compaction pressures, where the error bars represent the standard deviation (*n* = 5) and (**b**) pellet volume fractions, where each symbol represents an individual sample, the solid horizontal line represents the disintegration time of the control tablets, and the bracketing represents the standard deviation lines (dashed).

Tablets were prepared without pellets (control), with MCC pellets (15 mg in 200 mg tablet), and with XPVP pellets (15.5 mg in 200 mg tablet) at various compaction pressures and allowed to disintegrate, with the results depicted in Figure 5a. All disintegration times were below 8 s even at the highest compaction pressure of 40 MPa. The results show that the disintegration time of the MUPS tablets did not depend on the type of pellet core, as there was no significant difference between the disintegration times of tablets prepared with MCC or XPVP pellets at all four compaction pressures. Fitting trendlines to the results using the Tableau software revealed that the compaction pressure only increased the disintegration time significantly (*p* = 0.003) for the control tablets. The compaction pressure did not play a significant role in altering the disintegration time of MUPS tablets compacted with either MCC or XPVP pellets.

The disintegration times of the MUPS tablets fabricated with various numbers of pellets or pellet volume fractions were also determined (Figure 5b), and the results demonstrate that all MUPS tablets disintegrated in less than 8 s. Moreover, MUPS tablets with a higher pellet volume fraction generally disintegrated faster. Therefore, the disintegration results assure that the differences in disintegration time should not confound the MET release findings used to quantify pellet coat damage.

### 3.5. Dissolution

#### 3.5.1. Drug Release from Uncompacted Pellets

One objective of this study was to compare the performance between XPVP pellets and MCC pellets against pellet coat damage. The MDT_UC_ of the MCC and XPVP pellets were 26.1 ± 1.69 min and 28.7 ± 1.83 min, respectively. Although the MDT_UC_ of the XPVP pellets was slightly longer, the MDT_UC_ values were not significantly different (*p* = 0.082) between the two types of pellets. Nonetheless, subsequent MDT_C_ values were converted into K values to avoid any doubt regarding the extent of pellet coat damage between the MCC and XPVP pellets due to the slight difference in MDT_UC_.

#### 3.5.2. Effect of Compaction Pressure on Pellet Coat Damage

**Figure 6:**
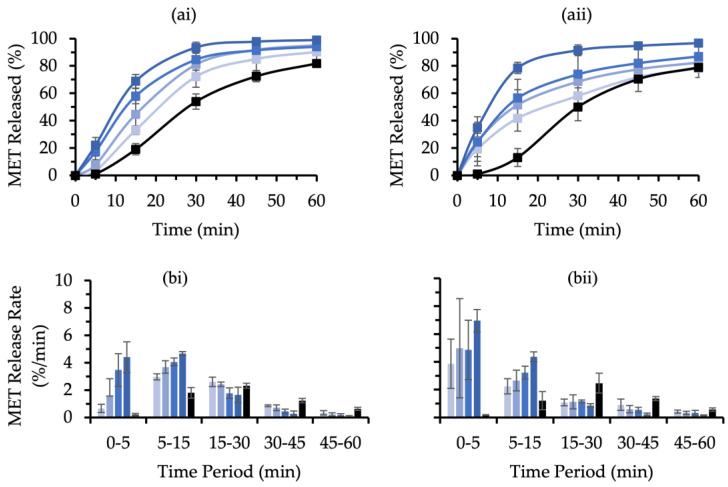
(**a**) MET released over time and (**b**) MET release-rate values per time period for (**i**) MCC and (**ii**) XPVP pellets compacted at 10 (∎), 20 (∎), 30 (∎), and 40 MPa (∎). Uncompacted pellets (∎) acted as control. Error bars indicate the standard deviation, and significant differences were omitted for clarity (*n* = 4).

An increase in compaction pressure is often needed to form stronger MUPS tablets, but this will consequentially increase the mechanical stress on pellet coats, which may adversely result in increased pellet coat damage. Ideally, good-quality coated pellets for MUPS tableting would have the capability to withstand a level of mechanical stress without suffering considerable pellet coat damage and impairment to its sustained release functionality. As such, MCC and XPVP pellets were tableted at various compaction pressures before being subject to a dissolution test. The MET release profiles for the MCC and XPVP pellets are shown in Figure 6(ai) and Figure 6(aii), respectively. The results show that higher compaction pressures led to faster MET release for both pellet types, thus alluding to EC pellet coat damage. At 10 MPa, the %MET released from the MCC pellets was significantly higher from that of the uncompacted MCC pellets across all time points (*p* < 0.05). For the compacted XPVP pellets, the MET release was only initially faster than the uncompacted XPVP pellets.

The MET release rates over several time periods are depicted in Figure 6b and provide a deeper insight into the sustained release function of the pellets. For the MCC pellets (Figure 6(bi)), the MET release rates correlated positively with the compaction pressure only during the 0 to 5 min (IDR) and 5 to 15 min time periods. For the XPVP pellets (Figure 6(bii)), there was no discernable correlation between the compaction pressure and IDR values. However, the MET release rates from the XPVP pellets during the 5 to 15 min time period correlated positively with compaction pressure. The IDR values of XPVP pellets compacted at 10 and 40 MPa were significantly higher (*p* = 0.034 and *p* = 0.012, respectively) than those from the MCC pellets. During the second time period of 5 to 15 min, the MET release rate from the XPVP pellets was significantly lower (*p* = 0.027) than that of MCC pellets. The MET release rates of compacted pellets fell below those of uncompacted pellets after 15 min. However, the MET release rates during the 15 to 30 min time period appeared to be much closer to those of the uncompacted pellets for the MCC pellets compared to the XPVP pellets.

**Figure 7:**
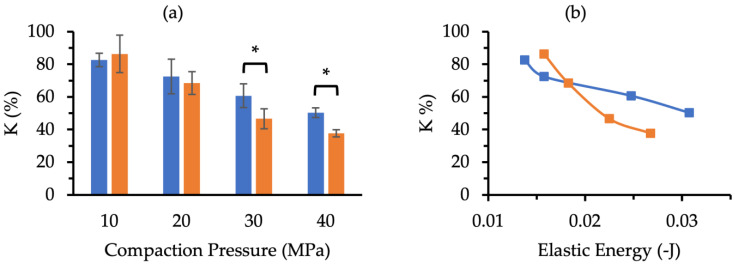
(**a**) K values of MCC (∎) and XPVP (∎) pellets tableted at various compaction pressures (*n* = 4). Error bars represent the standard deviation and * denotes a significant (*p* < 0.05) difference. (**b**) Plot of K values over tablet elastic energy where error bars were omitted for clarity (*n* = 4).

The MCC and XPVP pellets were compared further by determining the K values (Figure 7a) at each compaction pressure. At 10 and 20 MPa, the K values between the two pellet types were not significantly different (*p* > 0.05), whereas the K values of the XPVP pellets compacted at 30 and 40 MPa were significantly lower (*p* = 0.007 and *p* = 0.001, respectively) than those of the MCC pellets. A Tableau correlation analysis yielded trendline coefficients of −1.09%/MPa for the MCC and −1.68%/MPa for the XPVP pellets. These fitted trendlines had R^2^ values of 0.744 and 0.861 for the MCC and XPVP pellets, respectively (*p* < 0.0001 for both trendlines). The K values were plotted over the elastic energy values (Figure 7b). Since the tablet formulations were similar, except for the pellet used, the tablet elastic energy could provide an insight on the elastic recovery of the pellets in relation to the degree of pellet coat damage, and it appeared to correlate with K. It was also apparent that these correlations were slightly different for the MCC and XPVP pellets with a relatively steeper decline in K for the XPVP pellets.

#### 3.5.3. Effect of Pellet Volume Fraction on Pellet Coat Damage

**Figure 8:**
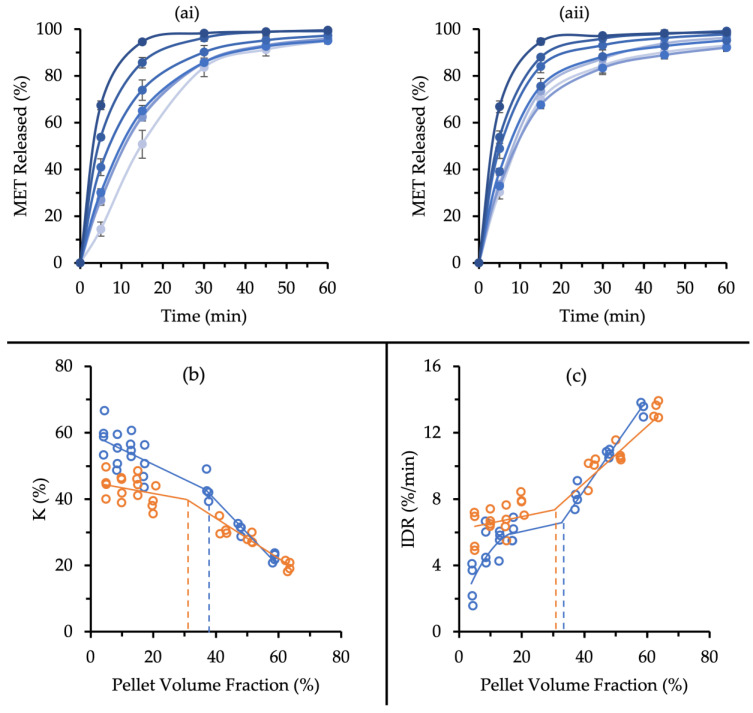
(**a**) MET released over time of (**i**) MCC and (**ii**) XPVP pellets compacted into MUPS tablets at 30 MPa with pellet volume fraction level I (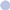), II (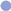), III (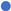), IV (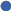), V (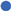), VI (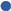), and VII (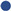). Error bars represent the standard deviation (*n* = 4). Plots of (**b**) K and (**c**) IDR values against pellet volume fractions for MCC (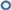) and XPVP (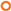) pellets. Each symbol represents an individual sample. Approximated trendlines and critical pellet volume fractions are shown for the respective pellets using solid and dashed lines, respectively.

Increased pellet volume fractions can increase the risk for pellet–pellet and pellet–tooling interactions that would exacerbate pellet coat damage. The effect of the pellet volume fraction on the degree of pellet coat damage was assessed by compacting MUPS tablets with various numbers of MCC or XPVP pellets at 30 MPa. This level of compaction pressure was selected because the MUPS tablet tensile strength values (Figure 2) at this level were approximately 1 MPa, which could be regarded as sufficiently strong tablets [59]. Subsequently, the MET release was measured and plotted over time to obtain the dissolution profiles (Figure 8a). The MET release was faster from MUPS tablets with higher pellet volume fractions for both pellet types.

The K values were determined from the dissolution profiles and plotted against the tablet pellet volume fractions (Figure 8b). The K values of the MCC pellets were generally higher than those of the XPVP pellets. A previous study evaluated the effects of increasing the pellet volume fraction and a critical pellet volume fraction of 39% was identified [40]. Below this critical pellet volume fraction, pellets were postulated to form a percolating, simple cubic lattice network that permitted increases in pellet volume fraction without much increase in coat damage. Above this critical fraction, pellet coat damage was found to increase steeply. The critical pellet volume fractions for the MCC and XPVP pellets were approximately 38% and 32%, respectively. The K values show that higher pellet volume fractions led to increased pellet coat damage for both the MCC and XPVP pellets. However, the XPVP pellets had slightly lower K values across all pellet volume fractions, particularly at the lower pellet volume fractions, indicating that the XPVP pellets experienced more coat damage than the MCC pellets. Moreover, the results suggest that the critical pellet volume fraction was slightly higher for the MCC pellets, thus suggesting that MUPS tablets with MCC pellets could be formulated with a slightly higher pellet volume fraction than XPVP pellets.

The IDR values were also determined and plotted against the pellet volume fractions (Figure 8c) to further compare the MCC and XPVP pellets. It appeared that the relationship between IDR and pellet volume fraction was similar to that of K. At increased pellet volume fractions, the IDR values increased for both pellet types. It was evident that IDR values were generally higher for the XPVP pellets. For MCC pellets, the IDR values at a pellet volume fraction of 5% were significantly lower (*p* = 0.03) than at 10%, whereas the K values were not different. Moreover, the critical pellet volume fractions using IDR as the indicator were approximately the same, at around 33% and 30% for the MCC and XPVP pellets, respectively.

## 4. Discussion

Extrusion-spheronization is a commonly employed technique in the production of drug-loaded pellets. Spheronization aids can be incorporated into the bulk material to enhance the formation of strong and spherical pellets [2,35,36,60]. Several materials have been identified and evaluated as spheronization aids in the literature. These include MCC, low substituted hydroxypropyl cellulose, kappa-carrageenan, hydroxypropyl methylcellulose, pectinate, and powdered cellulose [20,23,61,62,63,64,65]. Among the spheronization aids evaluated, MCC has been postulated to be the most effective due to its precise control and balance of water movement in and out of internal pores of the material [2]. However, MCC does have some limitations, and multiple alternative spheronization aids have been investigated. Such an alternative is XPVP, which has been successfully used as a spheronization aid to produce drug-loaded [60,66,67] or sacrificial-cushioning [43] pellets in MUPS tablets. Although its usefulness as a spheronization aid has been established, XPVP has not been studied well as a spheronization aid for drug-loaded pellets coated with a sustained release film compacted into MUPS tablets. Therefore, this study aimed to evaluate the suitability of XPVP as a replacement for MCC by comparing the performance of pellets spheronized using XPVP against pellets spheronized with MCC in terms of their resistance to pellet coat damage due to compaction.

The physical properties (Table 3) of the XPVP and MCC pellets differed significantly in several aspects. Firstly, the XPVP pellets had a larger aspect ratio, which is indicative of the XPVP pellets being more elongated. Secondly, the span of the XPVP pellets was higher, alluding to the pellet size distribution being greater. In general, pellets of a higher quality would have an aspect ratio near unity and a consistent size, which is indicated by a low span value [2]. Thirdly, the mean size of XPVP pellets (i.e., MPS) was generally higher, indicating that the use of XPVP resulted in larger pellets compared to MCC. A difference in pellet size may affect the drug release, as the surface-to-volume ratio may differ. This size difference may be adjusted by using an extrusion screen of smaller aperture size during pellet production [15,68,69]. However, the MDT_UC_ values were not significantly different. The K values calculated would nullify any difference in drug release due to differences in pellet size. Moreover, the MCC pellets had a higher crushing strength than the XPVP pellets, indicating that the MCC pellets were mechanically stronger. In MUPS tableting, it is preferred to have stronger pellets, as they would be more resistant to compaction-related pellet coat damage [5]. The pellet roundness and pellet weight were not significantly different. However, the slight difference in average pellet weight was considered when formulating MUPS tablets containing various numbers of pellets (Table 2). The results indicate that the quality of the MCC pellets was slightly better, which corroborates to MCC’s popularity as the spheronization aid of choice [2].

Previously, it was shown that the amount of water and the XPVP concentration used during extrusion-spheronization played a crucial role in the formation of pellets of comparable quality to MCC pellets [2]. In this study, these parameters were kept constant for the purposes of equal comparison between the two spheronization aids. XPVP yielded pellets that were reasonably good, albeit of slightly poorer quality compared to MCC. It could be possible to fabricate XPVP pellets with quality attributes more comparable to those of the MCC pellets by increasing and optimizing the water and XPVP concentration levels or by adjusting the extrusion-spheronization parameters. However, that was outside the scope of this work and could be included in a further study.

A sufficiently high compaction pressure is needed to form MUPS tablets of adequate tensile strength, with minimal capping, fractures, and friability [70]. The nature of the pellet core can affect the MUPS tablet tensile strength and disintegration time. For example, incorporating waxes into sacrificial cushioning pellets was found to mitigate pellet coat damage [71]. The disadvantages of this approach were the formation of weaker tablets and prolonged tablet-disintegration time [56,71]. Nevertheless, the MCC and XPVP pellets had different crushing strengths and may therefore have affected the MUPS tablet tensile strength (Figure 2) and disintegration time (Figure 5) differently. The results show that the inclusion of pellets generally weakened the MUPS tablets. Furthermore, despite the weaker XPVP pellets, there were few differences in tensile strength between the MUPS tablets containing MCC or XPVP pellets. Hence, the inclusion of pellets in the compact had a larger impact on the MUPS tablet tensile strength than the physical nature of the pellets. Nonetheless, it was evident that the formation of strong MUPS tablets may require a higher compaction pressure than control tablets. Furthermore, the disintegration times (Figure 5) were not significantly different for MUPS tablets containing either XPVP or MCC pellets. Most crucially, the disintegration times across all compaction pressures and pellet volume fractions were still very fast and should therefore not confound the MET release behavior from the coated pellets nor the use of dissolution tests for assessing the extent of pellet coat damage.

K values below 100% indicate an increase in drug release due to pellet coat damage. The K values (Figure 7a) demonstrate that both pellets experienced damage due to compaction. When compaction pressure was increased to 30 and 40 MPa, the XPVP pellets had significantly more coat damage than the MCC pellets. The pellet volume fraction in this evaluation was minimized to avoid risk factors that could affect pellet coat damage in MUPS tablets and thus could confound the direct effect of the compaction pressure. These risk factors include pellet–pellet interactions, pellet–tooling collisions, and pellet location [7]. The high R^2^ and low *p* values of the regression analysis support the validity of the models that describe the relationship of K with compaction pressure. The greater regression coefficient of the XPVP pellets indicates that the XPVP pellets experienced more damage per increase in compaction pressure. The MET release rate values (Figure 6b) further demonstrate that the XPVP pellet coat damage was generally higher and more variable. As more MET was released, the drug release rate consequently decreased and fell below that of corresponding uncompacted pellets, thus indicating a loss in sustained release function. The MET release rate results show that the MCC pellets retained more of the sustained release function than the XPVP pellets. The results also demonstrate that the XPVP pellets were more susceptible to damage with increased compaction pressure. Hence, altering the compaction pressure during the development of MUPS tablets would impart a greater effect on the pellet coat damage of XPVP pellets compared to MCC pellets.

The lower XPVP pellet crushing strength (Table 3) and steeper relationship between K and the elastic energy (Figure 7b) of MUPS tablets containing XPVP pellets explain why the XPVP pellets experienced more coat damage at higher compaction pressure than the MCC pellets. These results demonstrate that the weaker XPVP pellets were more prone to deformation and breaking. Consequently, the mechanical stress experienced by the EC coat would be higher, thereby resulting in greater XPVP pellet coat damage. As such, formulating MUPS tablets using XPVP pellets would potentially necessitate lower compaction pressures in conjunction with longer dwell times to increase tablet tensile strength without increasing pellet coat damage [5]. Furthermore, the use of XPVP pellets would require the incorporation of pellet cushioning agents to mitigate the pellet coat damage. Various cushioning agents have been evaluated in previous studies [43,71,72,73,74,75]. Future research could evaluate the effectiveness of these cushioning agents for XPVP pellets. Nevertheless, the MCC pellets were less susceptible to pellet coat damage than the XPVP pellets and would preferably be utilized.

MUPS tablets inherently provide formulators with an opportunity to adjust the drug dosage by altering the pellet volume fraction. However, increasing the pellet volume fraction beyond a critical limit could adversely increase the probability and severity of pellet–pellet and pellet–tooling interactions during compaction [7,53]. This would potentially result in an undesired increase in pellet coat damage. Understandably, high-performing pellets would be able to resist coat damage at higher pellet volume fractions. In this regard, MCC and XPVP pellets were compared by preparing MUPS tablets of different pellet volume fractions ranging from 5% to 65%. The dissolution profiles demonstrate that increased pellet volume fractions resulted in faster MET release due to pellet coat damage. As seen in Figure 8, the XPVP pellets generally had more pellet coat damage than the MCC pellets across the pellet volume fraction range. It was apparent that at equivalent pellet volume fraction levels (i.e., levels I to VII), the actual pellet volume fraction was slightly different between the two pellet types. This suggests that the physical nature of the pellet influenced the degree of tablet compression, with the XPVP pellets resulting in greater overall material compression, potentially because of their weaker nature, which permitted further pellet and tablet deformation. Additionally, the critical pellet volume fractions were approximated for the MCC and XPVP pellets. The MCC pellets appeared to have a higher critical pellet volume fraction (~38%) compared to XPVP pellets (~30%). Collectively, the results demonstrate that the XPVP pellets were more susceptible to pellet coat damage than MCC pellets when more pellets were incorporated into the MUPS tablet. Therefore, the formulation of MUPS tablets with high pellet volume fractions favors the use of MCC as a spheronization aid.

Increases in pellet volume fraction may impact MUPS tableting, and hence the effect of using various proportions of MCC or XPVP pellets on compaction energies was investigated (Figure 4). Both the pellet type and number (i.e., pellet volume fraction) affected the compaction energies. The rearrangement energy (Figure 4a) increased linearly with the number of pellets for both pellet types. This direct correlation suggests that for the same mass, pellets exhibit greater movement within the compact compared to filler particles. This effect could be attributed to the MCC filler particles succumbing to deformation (i.e., where rearrangement is no longer possible) relatively sooner than pellets. Due to their larger size and more spherical shape, the pellets would be able to slide around more, exhibiting relatively higher resistance against deformation and thus greater rearrangement energy. MUPS tablets containing MCC pellets had slightly higher rearrangement energies than those with XPVP pellets, which suggests that for the same energy applied to the filler–pellet mixture, more compression energy may contribute to plastic energy for XPVP pellets. Plastic energy (Figure 4b) reduced logarithmically with a greater number of pellets, likely because there was less MCC filler material present to deform plastically, whereas the proportion of relatively less plastically deformable pellets increased. The plastic energies of MUPS tablets with XPVP pellets were higher than those with MCC pellets, which shows that XPVP pellets plastically deformed more than MCC pellets. This supports the concept that the XPVP pellets deformed more plastically, which in turn contributed to their greater pellet coat damage at higher compaction pressures by the exertion of greater mechanical stress on the EC coat.

Compression energy (Figure 4c) also decreased logarithmically with a greater number of pellets, which indicates that tablets with higher pellet volume fractions were weaker. This could be attributed to the reduced presence of the filler particles and corresponding reduction in their contribution of good binding properties with an increased proportion of pellets. MUPS tablets containing XPVP pellets exhibited lower ejection energies (Figure 4d) at higher pellet volume fractions (levels V and above). This may be the only advantage of XPVP pellets over MCC pellets, as high ejection energy can lead to unwanted tablet issues such as cracking, lamination, or punch sticking [76,77].

Both the number of pellets contained within the MUPS tablet and their physical properties were found to significantly affect the MUPS tableting process. Hence, the impact of the pellet core and volume fraction on the degree of pellet coat damage and production of MUPS tablets should be carefully considered.

## 5. Conclusions

This study compared the performance of drug-loaded pellets prepared with XPVP or MCC as a spheronization aid against EC pellet coat damage induced by compaction. The MCC and XPVP pellets were prepared as MUPS tablets at various compaction pressures and at various pellet volume fractions. These tablets were then subjected to a dissolution test to quantify the extent of pellet coat damage, and the tablet compaction energies were also determined. The results demonstrate that the pellets spheronized with XPVP experienced relatively marginal but significantly more pellet coat damage and suggest that the critical pellet volume fraction was slightly lower for the XPVP pellets. The lower performance of the XPVP pellets was attributed to their lower crushing strengths, which made them more prone to coat damage during compaction. The mechanism of this effect was postulated to be greater pellet plastic deformation that raised the stress level on the EC pellet coat, thereby exacerbating coat damage. These findings provide formulators with important insights into the use of XPVP as a spheronization aid for the production of pellets and highlight the need for additional strategies to mitigate the pellet coat damage in MUPS tablets containing XPVP pellets.

## Figures and Tables

**Table 1:** EC coating process parameter settings.

Operating Condition	Setting
Atomizing air pressure (bar)	1.2
Inlet air temperature (°C)	60
Outlet air temperature (°C)	40
Nozzle-tip diameter (mm)	0.8
Nozzle-tip protrusion level (mm)	2
Spray rate (g/min)	8
Inlet air temperature (°C)	60

**Table 2:** MUPS tablet-preparation parameters to evaluate the pellet volume fraction.

Number of Pellets	MCC Pellet Weight (mg)	XPVP Pellet Weight (mg)	Intended Pellet Volume Fraction (%)	Pellet Volume Fraction Level
20	13.4	13.8	5	I
40	26.8	27.7	10	II
60	40.1	41.5	15	III
80	53.5	55.4	20	IV
160	107.0	110.8	45	V
200	134.0	138.0	60	VI
240	160.8	165.7	70	VII

**Table 3:** Physical properties of coated pellets. Mean values shown with the standard deviation.

Property	MCC	XPVP
Aspect ratio	1.15 ± 0.08	1.27 ± 0.15
Roundness	1.14 ± 0.08	1.15 ± 0.09
Pellet weight (mg)	0.67 ± 0.20	0.69 ± 0.16
D10 (mm)	1.04 ± 0.01	1.08 ± 0.01
D50 (mm)	1.13 ± 0.01	1.20 ± 0.03
D90 (mm)	1.24 ± 0.02	1.36 ± 0.04
Span	0.18 ± 0.01	0.23 ± 0.01
MPS (mm)	1.05 ± 0.04	1.14 ± 0.01
p_t_ (g/mL)	1.41 ± 0.03	1.20 ± 0.04
Crushing strength (MPa)	11.13 ± 3.15	5.59 ± 1.39

## Data Availability

Data presented in this study can be made available upon reasonable request.
